# Calcium Unified: Understanding How Calcium’s Atomic Properties Impact Human Health

**DOI:** 10.3390/cells14141066

**Published:** 2025-07-11

**Authors:** Karen B. Kirkness, John Sharkey, Suzanne Scarlata

**Affiliations:** 1 Health Professions Education Unit, Hull York Medical School, York YO10 5DD, UK; 2 Irish College of Osteopathic Medicine, National Training Centre, T23 C6P5 Cork, Ireland; john.sharkey@ntc.ie; 3 Department of Chemistry and Biochemistry, Worcester Polytechnic Institute, Worcester, MA 01609, USA; sfscarlata@wpi.edu

**Keywords:** calcium, coordination geometry, cell signaling, extracellular matrix, tensegrity, calcium-mediated tensegrity, basic sciences, biomedical education

## Abstract

Calcium plays a major role in all cellular functions, and its regulation is important in all aspects of human health. This key role calcium plays in cell function can be traced to its unique molecular coordination geometry, which is often overlooked in understanding calcium function. In this review, we integrate calcium’s ability to form various complexes with proteins and small molecules with its role as a key signaling atom. We argue that calcium’s ability to vary its coordination structures, compared to magnesium’s rigid geometry, explains its importance in biological functions. By examining calcium-mediated proteins, such as those containing EF-hand domains and those that assemble and stabilize the extracellular matrix in tissue organization, we demonstrate how calcium’s varied geometric coordination serves as both a signaling molecule and a regulator of physiological homeostasis.

## 1. Overview

Calcium stands at a unique intersection in biological systems, functioning simultaneously as a structural coordinator and signaling molecule across multiple scales [1,2]. While most investigators focus on calcium as a chemical messenger, it is also an important mediator of cellular and biological events across multiple scales. The reason underlying calcium’s broad impact is its distinctive coordination geometry, particularly its triangulated arrangements approximating Platonic solids (cubes, tetrahedra, octahedra, dodecahedra, and icosahedra).

As discussed below, this geometric property enables calcium’s versatility in biological systems, distinguishing it from other cations, notably magnesium [3]. Calcium’s coordination with proteins and their domains sets the stage for maintaining the 10,000-fold concentration gradient of Ca^2+^ across cell membranes—with extracellular calcium at approximately 1.2 mM and resting intracellular concentrations at an extraordinarily low 100 nM [1]. This steep gradient enables not only calcium’s role as a rapid second messenger but also underlies its capacity to function amongst distinct subcellular compartments [4] as a tensional mediator across biological scales [5].

## 2. Calcium’s Coordination Geometry: From Platonic Solids to Protein Recognition

### 2.1. Understanding Calcium Geometry

**Figure 1:**
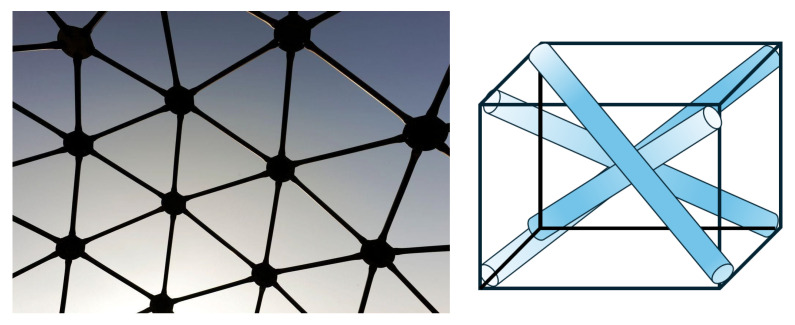
(**Left**)—A geometric architectural system composed of triangulated rigid elements that distribute stress across the structure, enabling lightweight yet highly stable designs through optimized load-bearing geometry. (**Right**)—A diagram of a cube where the internal rods generate a force opposite to the outside lines resulting in a stable cubic structure.

**Figure 2:**
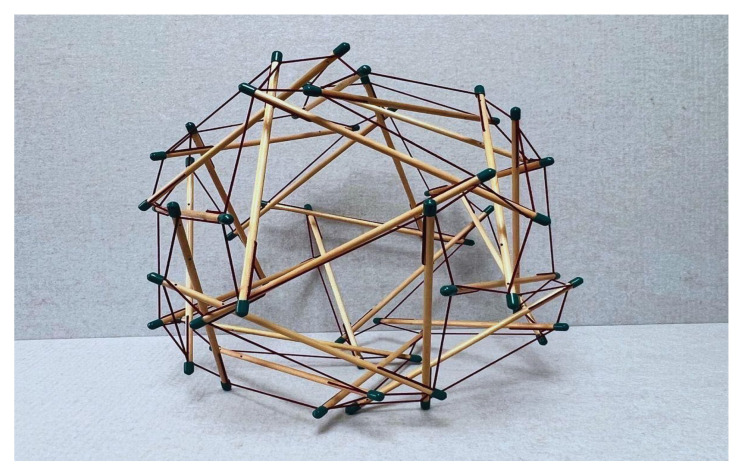
Prestressed (before loading) tensegrity structure that uses a triangulated or modular network of tension elements to stabilize isolated compression components, distributing mechanical forces through a balance of continuous tension and discontinuous compression.

To understand calcium’s ability to form varying coordination complexes in proteins, we first define tensegrity or “tensional integrity” [6]. Tensegrity is an architectural principle describing structures that stabilize their shape through a continuous network of pulling or stretching forces (i.e., tensile forces) balanced by discontinuous squeezing (compressive) elements. In its fundamental form, tensegrity refers to a structural system in which shape stability depends on how the entire structure distributes and balances mechanical stresses (see Figure 1 and Figure 2).

Key characteristics of tensegrity structures include (see Ingber [7]):

a. Continuous tension network: A network of elements under constant tension that forms the primary force-bearing system, noting that tensile elements require less energy for production and maintenance.
b. Discontinuous compression: Isolated rigid elements that resist compression but do not directly touch one another, allowing for porous arrangements rather than bulk solids that offer greater structural efficiency and versatility.
c. Self-stabilizing equilibrium: The structure maintains its integrity through a balance of opposing forces, creating prestressed mechanical stability.
d. Triangulation and prestress: Employs triangulated arrangements that efficiently distribute forces throughout the structure, providing both flexibility and strength, and offering complementary means to stabilize discrete networks.
e. Energy-minimizing efficiency: Achieves structural integrity with minimal material by optimizing force distribution.
f. Hierarchical organization: Tensegrity systems manifest across multiple scales, with smaller units serving as building blocks for larger structures. This nested hierarchy directly aligns with Ingber’s principle [7] that “structural efficiency is maximized and evolution accelerated through the use of hierarchical networks,” enabling emergent properties to arise at each level.

The principles of geodesic geometry (Figure 1), popularized by Fuller [6], are evident across biological systems, ranging from molecular to macroscopic scales. These triangulated networks represent nature’s solution for optimizing structural integrity through balanced force distribution across the scale hierarchy (Table 1)—the same principle observed in calcium’s coordination geometry.

### 2.2. The Metal That Moves Us

Calcium ions (Ca^2+^) play a central role in virtually every aspect of cellular function, serving as the most versatile and universally employed messenger in biological systems [1]. This ubiquity is not coincidental but reflects calcium’s unique coordination chemistry, which enables it to orchestrate interconnected processes that collectively implement tensegrity principles across biological scales. The interplay between Ca^2+^ signaling and mechanical forces allows cells to adapt to environmental changes and maintain structural integrity [4,5,8].

Biological systems have evolved to utilize calcium as a key mediator in cellular events, resulting in numerous proteins and enzymes whose physical and regulatory properties depend on changes in cellular calcium levels. The foundation of calcium’s biological versatility lies in its signaling capacity. As a universal second messenger, calcium encodes information through precisely modulated oscillations in frequency, amplitude, and spatial distribution, regulating diverse cellular activities from growth and division to survival and death [9,10]. This signaling does not occur in isolation but within an intricate homeostatic network spanning multiple cellular compartments—plasma membrane, endoplasmic reticulum, and mitochondria—where transporters, buffers, and channels ensure exquisite control over calcium concentrations and downstream effects [11,12,13].

### 2.3. Calcium as a Second Messenger in Cellular Signaling

#### 2.3.1. Calcium’s Physical Chemistry in Biological Systems

Calcium ions (Ca^2+^) function as universal second messengers, translating external stimuli into precise cellular responses through a hierarchical signaling cascade where the first messengers (hormones, neurotransmitters) activate membrane receptors, triggering intracellular Ca^2+^ release or influx that coordinates downstream processes, including excitation-contraction coupling, exocytosis, and endocytosis (see Table 2).

Ionized vs. Bound Calcium: Only free ionized Ca^2+^ (~50% of total calcium) contributes to biological action, while bound calcium serves as a readily mobilizable reservoir. The +2 charge enables calcium’s characteristic coordination geometry as previously described, creating the geometric versatility that allows coordination with negatively charged amino acid residues while maintaining the spatial flexibility required for protein conformational changes. Calcium ions can coordinate with 6–8 ligands, adopting geometries from octahedral to pentagonal bipyramidal that accommodate different binding site shapes and environments through flexible interactions with negatively charged phospholipid head groups where it can stabilize lipid bilayers and negatively charged residues, such as aspartate and glutamate [1,2,14,15].

This coordination geometry enables calcium binding to trigger significant conformational changes in proteins, particularly those with EF-hand motifs where calcium binding reorganizes helical packing and opens domains to enable target protein interactions—as seen in calmodulin and troponin C—or prepares proteins for activation, like gelsolin’s actin-binding capability [16,17]. The +2 charge thus provides the optimal balance of binding strength and reversibility, ensuring that calcium’s geometric coordination can induce and stabilize the conformational changes essential for diverse cellular functions while remaining sensitive to the local protein environment.

**Figure 3:**
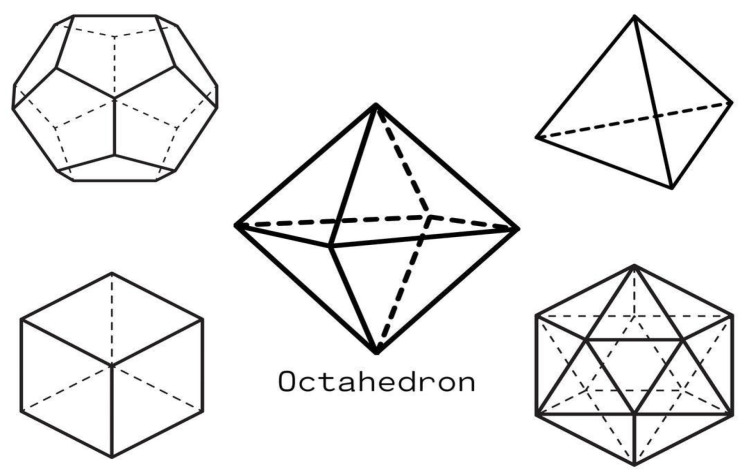
The octahedron represents one of the primary coordination geometries for Ca^2+^ ions in biological systems. This Platonic solid consists of 6 coordination points arranged symmetrically around the central calcium ion, with each point equidistant from the center. The resulting structure has 8 triangular faces, creating perfect triangulation at each face.

Hydration and Thermodynamics: Calcium’s exothermic hydration creates a dynamic water shell that adopts the same flexible coordination patterns (6–8 water molecules in geometric arrangements approximating Platonic solids) (see Figure 3). This hydrated coordination sphere can readily exchange water molecules for protein ligands while preserving the triangulated geometric relationships that define calcium’s binding specificity and enable rapid, reversible protein interactions essential for signaling [18,19]. This flexible “coat” of water molecules arranged in precise geometric patterns enables calcium to function as biology’s universal signaling ion. When calcium needs to bind to proteins, it can quickly replace these water molecules with protein components while maintaining its overall shape. This flexibility allows calcium to rapidly switch between different proteins for cellular signaling.

The exothermic nature of calcium-water interactions stabilizes the coordination geometry while maintaining sufficient flexibility for ligand exchange—water molecules are rapidly replaced by oxygen or nitrogen atoms from protein side chains (carboxyl groups of aspartate and glutamate, amide groups) without disrupting the core geometric framework [19,20]. This dynamic exchange process is spectroscopically observable as shifts in protein infrared spectra during calcium binding, reflecting local environmental changes as the hydration shell adapts to accommodate protein ligands while preserving calcium’s characteristic coordination patterns that enable its versatile biological functions [19,21].

Cation Competition: Calcium’s intermediate charge density (12.6 C/mm^3^) and large ionic radius (114 pm) create optimal geometric complementarity with protein binding sites designed for flexible coordination numbers. Unlike magnesium’s rigid octahedral geometry (86 pm, 23.3 C/mm^3^) or sodium’s loose coordination, calcium’s geometric adaptability provides the precise balance of binding strength and reversibility essential for dynamic signaling cascades (see Table 3). Despite the higher charge density of competing ions like Mg^2+^, calcium-binding proteins selectively bind Ca^2+^ due to geometric constraints and many-body polarization effects, where the energetic cost of packing smaller, more highly charged ions into flexible protein sites favors calcium’s intermediate properties [14,15,22].

#### 2.3.2. Signal Initiation and Propagation

Understanding calcium’s unique geometric properties and how its flexible “water coat” enables rapid protein binding sets the stage for cellular action, as Ca^2+^ is a central regulator in both excitation-contraction coupling (ECC) in muscle and in the processes of endo- and exocytosis in excitable cells. Extracellular stimuli activate membrane receptors (not calcium itself) [9], triggering Ca^2+^ influx through voltage-gated channels or release from intracellular stores via IP_3_ and ryanodine receptors. This elevation orchestrates excitation-contraction coupling in muscle cells, vesicle fusion during exocytosis, and membrane retrieval in endocytosis through the coordination of calcium with regulatory proteins such as troponin, synaptotagmin, and calmodulin. A network of pumps, exchangers, and buffers maintains precise calcium gradients, preventing cytotoxicity while enabling signal amplification where single first messenger molecules trigger the release of thousands of calcium ions.

Receptor activation launches signaling pathways that open specific calcium channels, particularly inositol 1,4,5-trisphosphate (IP_3_) receptors and ryanodine receptors, generating localized calcium microdomains or propagating calcium waves throughout the cell [23,24]. These spatiotemporally complex Ca^2+^ signals are decoded by diverse calcium-binding proteins that undergo conformational transformations upon calcium coordination [25]. These structural shifts enable interactions with downstream effector molecules, orchestrating essential processes from synaptic vesicle exocytosis to sarcomere shortening [10].

IP_3_ and Ryanodine Receptors: Coordination Chemistry Architecture: The two primary intracellular calcium release channels—IP_3_ receptors (IP_3_R) and ryanodine receptors (RyR)—represent evolutionary masterpieces of calcium coordination engineering that serve as central coordinators of intracellular calcium release in response to both mechanical and electrical stimulation. These massive tetrameric proteins exemplify how calcium’s coordination geometry enables exquisitely precise signaling responses through their shared architectural blueprint yet distinct regulatory personalities, functioning as key molecular links between external cues and intracellular calcium dynamics that underpin movement, muscle metabolism, and mechanotransduction [26,27].

Calcium Binding Affinities and Activation Mechanisms: These channels operate through sophisticated activation mechanisms: mechanical forces in specialized cells like enteroendocrine cells activate mechanosensitive channels (Piezo2), triggering initial calcium influx that recruits IP_3_R and RyR for signal amplification supporting hormone secretion and epithelial function, while electrical depolarization in muscle cells induces coordinated calcium release from endoplasmic or sarcoplasmic reticulum essential for contraction and downstream signaling pathways regulating gene expression and metabolism [28,29].

IP_3_ Receptor Affinity Data and Biphasic Regulation: IP_3_ receptors showcase calcium’s biphasic mastery—a molecular Jekyll and Hyde activated by inositol 1,4,5-trisphosphate binding (Kd ≈ 10–100 nM) yet governed by calcium’s own dual nature (where the same ion that activates the channel at low concentrations becomes its inhibitor at high concentrations). Through multiple EF-hand-like domains and strategically positioned acidic residues, these channels demonstrate calcium coordination’s precision: gentle activation at low concentrations (EC_50_ ≈ 200–300 nM) transforms into firm inhibition at higher levels (IC_50_ ≈ 10–20 μM). This is coordination chemistry’s elegant solution for spatial and temporal signal control [30].

The channels’ allosteric sophistication demands that all four IP_3_ binding sites be occupied for opening—a molecular democracy where consensus rules [31]. Meanwhile, their functional cross-talk creates calcium signaling symphonies: RyR-mediated calcium-induced calcium release amplifies IP_3_R-triggered signals, shaping everything from muscle contraction to gene expression through geometric precision [32,33].

Ryanodine Receptor Calcium Sensitivity and EF-Hand Coordination: Similarly, ryanodine receptors demonstrate exquisite calcium sensitivity through their EF-hand domains, with RyR1 showing calcium activation (EC_50_ ≈ 1–5 μM) and calcium-induced calcium release that depends on the geometric arrangement of multiple calcium-binding sites. These EF-hand domains function as paired conformational switches that interact allosterically with adjacent channel regions—particularly the S2-S3 loop—creating a molecular toggle that transforms small calcium concentration changes into dramatic channel state transitions [34,35,36].

What makes RyR1’s calcium sensitivity germane to this review is how its dual EF-hand motifs orchestrate both activation and inactivation through spatial geometry alone. The precise three-dimensional arrangement ensures that calcium binding propagates conformational waves through the massive tetrameric structure, enabling rapid signal amplification while maintaining tight regulatory control [37]. This is evidence of molecular recognition converting into mechanical action. Isoform-specific differences between RyR1 and RyR2 further demonstrate how subtle variations in EF-hand geometry can fine-tune calcium responsiveness for specialized cellular contexts, from muscle contraction to cardiac rhythm regulation [38,39].

Coordination Geometry Principles in Channel Function: These affinity relationships directly reflect the octahedral and coordination flexibility principles underlying calcium’s biological versatility, where calcium’s ability to adopt geometries close to pentagonal bipyramidal or octahedral arrangements with coordination numbers typically ranging from 6 to 8 enables precise geometric complementarity between calcium ions and diverse protein binding sites. This geometric adaptability allows calcium to coordinate primarily with oxygen atoms from amino acid side chains and water molecules in flexible arrangements that support both high-affinity and low-affinity binding sites, enabling the switch-like behavior essential for intracellular signaling cascades [18].

The functional consequence of this coordination flexibility is calcium’s capacity to bind a wide array of proteins with different conformations and functions—from structural roles in biomineralization to dynamic roles in cell signaling and enzyme regulation—making it the universal signaling ion capable of mediating precise and adaptable cellular responses across diverse biological contexts [10,40,41,42,43,44].

#### 2.3.3. Changes in Protein Conformation and Functional Outcomes

The coordination of Ca^2+^ by target proteins induces precise structural rearrangements that modulate their activity, providing rapid and reversible regulation of cellular functions. In neurons, calcium influx modulates synaptic strength and neurotransmission, while in muscle tissue, calcium binding to troponin C initiates contraction by altering protein conformation and tension states [10,23,40]. The spatial distribution and temporal dynamics of calcium signals—orchestrated through the coordinated action of buffers, pumps, and exchangers—ensure these processes remain tightly regulated and fully reversible [23,40].

Beyond neurotransmission and contraction, calcium signaling governs cellular proliferation, differentiation, metabolism, and programmed cell death pathways including apoptosis and autophagy [10,40,41] (see Table 4). Disruptions in calcium homeostasis or signaling networks contribute to numerous pathological conditions, including neurodegenerative disorders, cardiovascular disease, and cancer progression, highlighting the critical importance of precise calcium regulation for cellular and organismal health [10,40,41,42,43,44].

#### 2.3.4. Ca^2+^ Is Equally Attuned to Mechanical First Messengers

Beyond its role in chemical signaling, Ca^2+^ serves as a critical mechanical signal transducer with profound implications for movement-based therapies. Through specialized mechanosensitive channels like Piezo proteins, mechanical stimuli directly trigger Ca^2+^ influx that initiates cascading cellular responses [45]. Mechanotransduction—the process by which cells convert mechanical stimuli into biochemical signals via calcium pathways—fundamentally shapes tissue function across multiple dimensions.

Mechanical forces sensed through receptors like integrins trigger calcium-dependent signaling cascades that modulate fibroblast behavior, directing extracellular matrix (ECM) synthesis, degradation, and reorganization to influence tissue architecture and mechanical properties [46,47]. The ECM itself functions as a “mechanical memory-storage device,” where sustained structural changes perpetuate altered tissue behavior long after the initial stimulus [48]. These same pathways regulate local inflammatory responses, where calcium-mediated tensional homeostasis influences immune cell function and pathogen responses [49,50].

Critically, mechanical forces transmit from the ECM through the cytoskeleton to the nucleus, where they alter chromatin structure and gene transcription, effectively recalibrating proprioceptive feedback mechanisms [51,52]. Perhaps most fascinatingly, this process creates a form of cellular “mechanical memory” through epigenetic modifications that encode past mechanical experiences, influencing future cell behavior, differentiation trajectories, and tissue adaptation capacities [53,54].

Understanding how different mechanical inputs—from sustained stretches to oscillatory mobilizations—generate specific calcium signaling patterns provides a mechanistic explanation for therapeutic efficacy beyond simplistic mechanical models [55,56]. This integrated calcium-centered framework bridges traditional disciplinary boundaries between anatomy, physiology, and biomechanics, offering practitioners a coherent explanation for how hands-on interventions propagate through biological systems to create lasting functional changes.

#### 2.3.5. ECM ↔ Ca^2+^: A Dynamic Reciprocity

What makes Ca^2+^ particularly significant for tensegrity is its bidirectional relationship with the cytoskeleton. Ca^2+^ regulates microtubule-associated proteins and actin remodeling, which are essential for cell shape, migration, and division [57,58]. Meanwhile, the cytoskeleton itself modulates calcium oscillations, creating a cyclical feedback that integrates external and internal cues [59]. Through this reciprocal interaction, Ca^2+^ influences the cytoskeleton’s generation of internal prestress—the balanced tension and compression that defines tensegrity at the cellular level [57,58].

This Ca^2+^-mediated tensional network extends beyond the cytoskeleton to membrane dynamics, where Ca^2+^ signaling coordinates formation and remodeling processes crucial for exocytosis, endocytosis, and selective engulfment [1]. During cell division, Ca^2+^-mediated cytoskeletal changes and membrane interactions link mechanical and chemical signals to ensure proper cellular expansion and separation [9,58]. Even cellular defense and homeostasis rely on Ca^2+^-dependent pathways that regulate autophagy and apoptosis, enabling selective component elimination [10,12].

The interplay between Ca^2+^ signaling, cytoskeletal prestress, and membrane dynamics creates an integrated tensegrity system that allows cells to maintain structural integrity while adapting to mechanical and chemical stimuli [57,58,59]. Remarkably, these mechanisms operate coherently from molecular to tissue levels, supporting coordinated responses to environmental and developmental cues across biological scales [9,58].

As detailed below, Ca^2+^’s coordination chemistry underpins a cyclical network of signaling, cytoskeletal regulation, membrane dynamics, and selective cellular processes that collectively implement tensegrity principles throughout living systems. This Ca^2+^-orchestrated tensegrity network enables cells to integrate mechanical and chemical information, maintain structural integrity, and adapt across biological scales—making Ca^2+^ not merely a signaling molecule but a fundamental coordinator of biological architecture.

### 2.4. Coordination Preferences and Geometric Complementarity

Understanding this biological architecture involves examining the geometric relationship between Ca^2+^ and proteins, specifically the entanglements known as coordination complexes. Ca^2+^ achieves these by binding to multiple electron-donating groups (typically oxygen atoms from proteins or water molecules), creating geometric arrangements that often approximate Platonic solids [60] (see Figure 3). This geometric resonance between molecular arrangements and mathematical forms reflects fundamental physical laws governing electron distribution and energy minimization.

With its relatively large ionic radius of approximately 100 picometers, Ca^2+^ frequently adopts octahedral coordination with 6 ligands arranged symmetrically to create a highly stable structure with triangulated faces [61]. This arrangement embodies perfect triangulation, with each face forming an equilateral triangle that maximizes stability while permitting mobility at the vertices [62].

**Figure 4:**
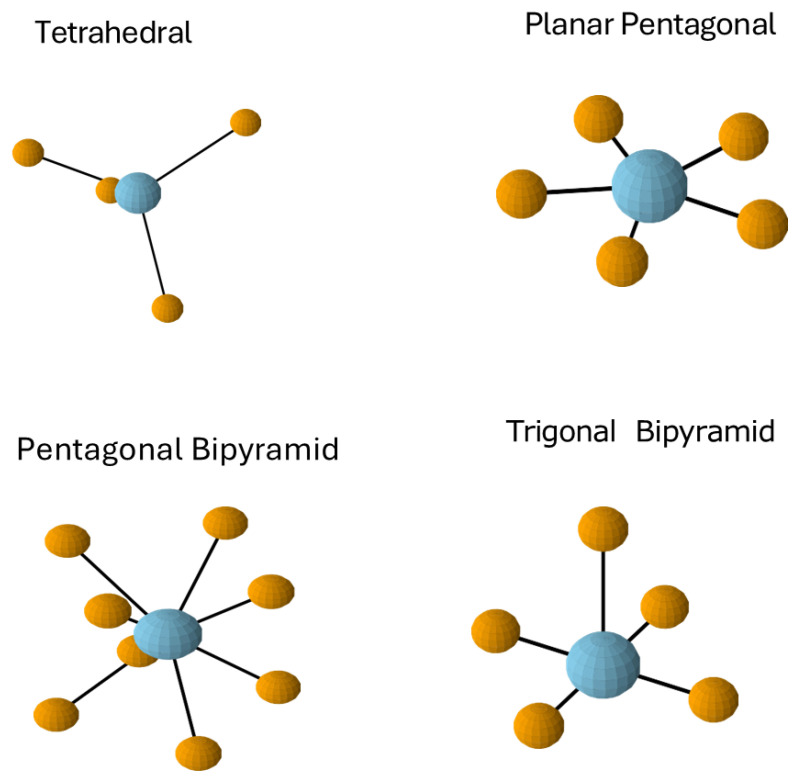
Different calcium coordination geometries found in proteins where calcium ions are in blue. These different coordination states can easily shift depending on the number and distance of available ligands. While calcium (blue ball) often forms octahedral (6-coordinate) complexes, its larger ionic radius and coordination flexibility allow it to accommodate 7–8 ligands (orange balls) in arrangements such as the pentagonal bipyramid.

Beyond octahedral geometry, Ca^2+^’s coordination flexibility encourages it to form extended di-coordination complexes with 7–8 ligands, creating more complex structures like pentagonal bipyramids [63], as illustrated in Figure 4. This flexibility distinguishes Ca^2+^ from many other biological cations and contributes to its versatile signaling functions.

### 2.5. The EF-Hand Motif

**Figure 5:**
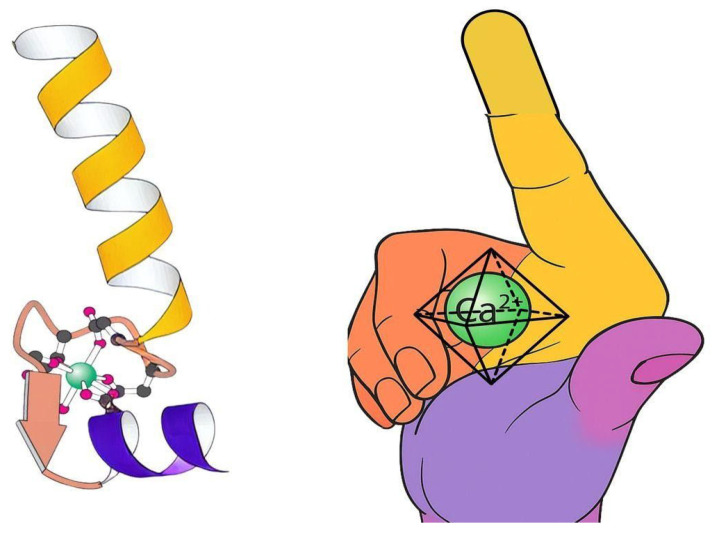
The EF-hand calcium-binding motif. (**Left**) The structural arrangement shows two alpha helices (E and F) connected by a calcium-binding loop. Specific amino acid residues position oxygen atoms to precisely coordinate the calcium ion. (**Right**) The hand-like structure where helix E represents the forefinger (yellow), the calcium-binding loop forms the middle finger (orange), and helix F forms the thumb (purple), with calcium binding in the “palm” region. (Adapted from Kretsinger, [25]).

Proteins have evolved to create geometric spaces that perfectly match Ca^2+^’s coordination preferences [64]. The EF-hand motif, consisting of two alpha helices (E and F) connected by a Ca^2+^-binding loop, illustrates nature’s precision engineering (see Figure 5). This motif positions oxygen atoms with extraordinary accuracy to coordinate Ca^2+^ ions in specific geometric arrangements [65]. The dramatic conformational change triggered by calcium binding demonstrates the lock-and-key relationship between calcium’s geometry and protein function, directly connecting spatial organization to biological signaling through Ca^2+^’s ability to trigger cascades [66].

## 3. The EF-Hand: Geometric Specialization in Calcium Signaling

### 3.1. The EF-Hand: Nature’s Calcium-Specific Sensor

The EF-hand motif represents an evolutionary masterpiece of geometric specialization for calcium binding [67,68]. Found in proteins like calmodulin (CaM, a universal signal transducer), this motif consists of two alpha helices (E and F) connected by a calcium-binding loop that positions oxygen atoms with extraordinary precision to coordinate calcium ions.

Calcium binding induces dramatic conformational changes in EF-hands that propagate through the protein structure, activating downstream molecular targets. This mechanism translates calcium coordination geometry directly into biological function—a compelling visual lesson in geometric signal transduction that helps students understand how spatial arrangements govern protein function. In this way, the triangulated coordination geometry of calcium serves as a unifying principle that connects atomic-scale interactions to macroscopic biological properties.

### 3.2. Functional Consequences of Coordination Geometry

The contrast between calcium and magnesium coordination provides further insight into the importance of these geometric relationships, with quantitative differences explaining their distinct biological roles [69]. While both are divalent cations, their physical properties diverge significantly. Magnesium’s smaller ionic radius drives it to form tighter, more rigid octahedral complexes with a strict coordination number of 6 and rapid water exchange kinetics (τ ≈ 10^−9^ s). In contrast, calcium’s larger size and lower charge density enable more flexible coordination with numbers ranging from 6 to 8 ligands, slower exchange rates (τ ≈ 10^−8^ s), and geometric versatility spanning octahedral to pentagonal bipyramidal arrangements. Recent research confirms these fundamental differences: magnesium’s hexaaquated complex ([Mg(H_2_O)_6_]^2+^) maintains rigid octahedral geometry with collective water rearrangement required for exchange, while calcium exhibits flexible coordination with several shallow local minima in its free-energy profile, reflecting its geometric versatility and weaker ligand binding that enables diverse protein binding environments [70,71,72].

These fundamental differences in binding affinity and coordination flexibility (i.e., magnesium favoring tight, static binding versus calcium’s adaptable, dynamic interactions) directly determine their respective biological functions: structural stabilization versus signaling versatility (see Table 3). Magnesium has a compact, rigid structure. Its strict adherence to perfect octahedral form makes it ideal for maintaining precise structural integrity, particularly in stabilizing nucleic acids through extensive hydrogen bonding networks [73] In contrast, calcium’s adaptable coordination (allowing 6-8 ligands) enables it to shift between geometric forms, making it ideal for triggering dynamic responses KREBS & H [74].

This geometric distinction has functional consequences. Magnesium behaves like a stone in the stream, anchoring and stabilizing the molecular terrain. At the same time, calcium resembles the water itself, embodying gesture and transition, fluid, responsive, and capable of reshaping the path it travels. These elemental metaphors capture more than symbolism; they reflect fundamental tendencies in how each ion engages with biological form and function. In biomineralization, magnesium’s compact coordination can interrupt crystallization, keeping minerals in an amorphous state POLITI [75], while calcium ions play a crucial role in DNA structure through their flexible coordination properties, bringing DNA strands closer together by reducing electrostatic repulsion between phosphate backbones XU [76]. These distinct coordination geometries translate directly to different biological functions with clinically relevant outcomes. Grabarek [77] noted that “some pathological conditions attributed to Mg^2+^ deficiency might be related to excessive activation of underlying Ca^2+^-regulated cellular processes,” highlighting the clinical relevance of these geometric distinctions.

## 4. Calcium as a Helical Mediator

### 4.1. Calcium’s Biological Role Through the Lens of Tensegrity

Calcium’s unique coordination geometry establishes it as a fundamental mediator of tensional forces across biological scales [78,79,80,81,82,83]. The pioneering cellular tensegrity research was first established by Ingber [84] and calcium coordination studies converge on a unified framework where calcium’s geometric properties directly influence mechanical tension at multiple scales.

In Ingber’s tensegrity model, cells maintain mechanical stability through a prestressed network of tensional microfilaments and compressional microtubules [85], which is conceptually like the tensegrity icosahedron shown in Figure 2. Calcium ions function as key regulators of this tensional network by modulating myosin light chain phosphorylation, directly affecting cytoskeletal prestress and mechanical responsiveness [86].

Calcium’s regulatory function is particularly evident in muscle contraction, where these ions orchestrate the precise molecular choreography of excitation-contraction coupling. Calcium coordinates myosin-actin bridging, protein synthesis, and degradation essential for muscle function [79,81]. The kinetics of calcium binding and release directly controls tension development in muscle fibers, with specific effects on ATPase activity and mechanical force generation [87,88]. Recent research extends this tensional role beyond the muscle, demonstrating calcium’s involvement in regulating membrane tension, which influences cellular morphology and neuronal plasticity [83].

Central to all these processes is calcium homeostasis—a ubiquitous regulatory system that maintains precise calcium gradients across biological scales. At the cellular level, mitochondria function as dynamic calcium reservoirs, directly linking energy metabolism to mechanical stability [89]. A coordinated network of ion channels and transporters maintains these critical calcium gradients that are essential for both structural integrity and mechanical function [90].

This homeostatic system extends dramatically across scales to encompass the entire skeletal system, where bones function as the body’s macroscopic calcium reservoir, storing 99% of the body’s calcium in a dynamic mineral phase. The same triangulated coordination geometry that governs calcium’s atomic interactions in proteins also determines the crystalline structure of hydroxyapatite in bone tissue, establishing a direct geometric connection between nanoscale coordination and anatomical structures [91].

Calcium release from bone occurs under several physiologically significant circumstances. Most notably, when serum calcium levels drop below the normal range (hypocalcemia), the parathyroid glands release parathyroid hormone (PTH), which upregulates osteoclast differentiation and activity to liberate calcium from the bone mineral matrix. This calcium mobilization also occurs during pregnancy and lactation, when maternal calcium demands increase dramatically to support fetal skeletal development and milk production. Estrogen withdrawal during these states enhances bone resorption.

Prolonged mechanical unloading, as observed in extended bed rest, spinal cord injury, or microgravity environments, provides a compelling demonstration of calcium-mechanical force dynamic reciprocity. When mechanical stimulation decreases, mechanosensitive osteocytes detect this reduction and initiate a cascade of signaling events that fundamentally alter bone metabolism [92,93]. This detection system exemplifies bidirectional calcium-mechanical communication: osteocytes sense mechanical strain through calcium-dependent mechanotransduction, and their response modulates calcium homeostasis at the organism level.

The molecular mechanisms underlying this process reveal the exquisite integration of mechanical and chemical signals. Osteocytes respond to reduced loading by increasing the RANKL/OPG ratio, which promotes osteoclastogenesis and subsequent bone resorption [94]. Simultaneously, these cells upregulate sclerostin production, which antagonizes Wnt/β-catenin signaling pathways essential for osteoblast activity and bone formation [93]. These molecular shifts collectively decrease bone density while liberating calcium into circulation.

Perhaps most fascinating is the bone matrix itself, which functions as a mechanochemical transducer. Direct mechanical stimulation of the mineralized matrix triggers measurable calcium efflux that can stimulate nearby osteoblasts, independent of cellular activity [95,96]. This matrix-level calcium release establishes a mechanical-chemical information pathway in which physical forces directly influence local calcium concentration, thereby affecting cellular behavior. Experimental models consistently demonstrate that unloading leads to quantifiable increases in calcium elimination from bone tissue [92,97], establishing a direct link between mechanical force, calcium signaling, and structural adaptation.

These pathways illustrate how calcium serves as both a sensor and an effector in a continuous feedback loop connecting the mechanical environment to skeletal adaptation—the quintessential example of dynamic reciprocity between physical forces and biological signaling across multiple scales of organization. The hierarchical calcium regulatory system exemplifies scale-spanning tensegrity principles, as the same fundamental calcium coordination geometry establishes baseline conditions necessary for tensional homeostasis across atomic to anatomical dimensions. This heterarchical relationship helps conceptualize how a single element maintains structural and functional continuity across twelve orders of magnitude in biological systems.

### 4.2. The EF-Hand as a Helical, Molecular Tension System

Moving from cellular to molecular scales, we return to the EF-hand motif—a quintessential example of how calcium directly mediates molecular tension. These specialized protein domains represent nature’s evolution of calcium-responsive tensegrity elements at the nanoscale, serving as the fundamental building blocks for larger tensional networks.

The EF-hand motif functions as a molecular tension system where calcium ions stabilize alpha-helical structures, creating what can be conceptualized as “molecular springs” [98,99]. These helical elements store and release energy during calcium-induced conformational changes, efficiently converting chemical signals to physical forces [100].

Research on S100A5 protein offers concrete evidence of calcium’s structural specificity, demonstrating how it maintains alpha-helical structure even when competing with other metal ions [101]. This selectivity illustrates fundamental principles of ion coordination geometry and its role in structural stabilization. Similarly, parvalbumin studies reveal how modifications to EF-hand motifs simultaneously alter both calcium binding affinity and alpha-helical content [102], establishing a direct relationship between coordination chemistry and mechanical function.

**Figure 6:**
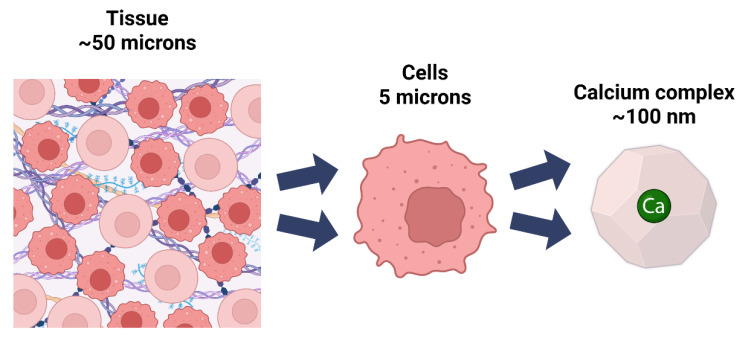
Cartoon showing the scales of calcium impact from ECM in tissues, to cells to calcium coordination complexes.

These EF-hand domains function as calibrated tension mediators, with their pre-tensed helical elements responding to calcium binding with precisely tuned mechanical adjustments [103]. The mechanical consequences of these molecular events directly influence larger cellular structures, including neuronal membrane properties and synaptic function [83,104] thus linking atomic-scale coordination geometry to cellular function through tensegrity (see Figure 6). 

Ca^2+^ plays a crucial structural role in the ECM by inducing conformational changes, bridging protein domains, and organizing supramolecular assemblies that effectively link molecular structure to tissue integrity. The concept of “prestress” in soft tissues, a cornerstone of Ingber’s tensegrity model, is directly mediated by calcium through these mechanisms [105]. By forming bridging complexes, stabilizing protein folding, and regulating mechanotransduction, calcium translates chemical principles into the physical forces that cells experience within tissues.

The tensional effects of calcium extend to cellular behavior within the ECM and a host of clinically relevant outcomes (see Table 5). Extracellular calcium concentration directly affects cell stiffness, adhesion, and migration capabilities, providing tangible examples of mechanobiology principles critical for understanding tissue repair mechanisms [106]. Moreover, disturbances in calcium homeostasis within the ECM drive numerous pathological conditions through disruption of the tensional balance.

Elevated extracellular calcium can induce pathological mineralization in vascular smooth muscle cells, leading to vascular calcification which is a key feature in atherosclerosis, aortic stenosis, and chronic kidney disease [107,108]. These processes involve the formation of matrix vesicles enriched in calcium-binding proteins that alter the ECM’s tensional properties. In articular cartilage, calcium imbalances contribute to crystal deposition diseases and osteoarthritis by disrupting the normal tensegrity-based mechanical properties of the tissue [109]. Perhaps most illustrative of calcium’s structural role are genetic mutations affecting calcium-binding sites in ECM proteins such as fibrillin, causing connective tissue disorders like Marfan syndrome that manifest as disruptions in tissue tensional integrity [110].

Calcium’s regulatory functions extend to immune responses, where high extracellular calcium in rheumatoid arthritis drives macrophage differentiation and amplifies joint inflammation [111]. The calcium-sensing receptor (CaR) serves as a critical regulator of this extracellular calcium tensional network, with its dysfunction resulting in disorders of calcium metabolism [112]. Disruptions to calcium’s coordination geometry at the molecular level can cascade to tissue-scale mechanical dysfunction.

Extracellular calcium concentration directly affects cell stiffness, adhesion, and migration capabilities, providing tangible examples of mechanobiology principles critical for understanding tissue repair mechanisms [106]. Similarly, calcium ion diffusion through the brain’s ECM illustrates biophysical principles of signal propagation—creating natural interdisciplinary connections between neurochemistry, physics, and physiology [113,114].

Fibronectin offers a particularly instructive case study in calcium-mediated mechanochemistry. When mechanical tension is applied to fibronectin fibers, cryptic calcium-binding sites are exposed, triggering fibrillogenesis along tension field lines [115]. This process elegantly demonstrates the bidirectional relationship between physical forces and calcium-mediated biochemical responses, illustrating how mechanical information is transduced through calcium coordination.

## 5. Discussion: Bridging Geometry and Biology

### 5.1. Calcium’s Impact Across Multiple Scales

This narrative review reveals how calcium’s coordination geometry embodies a deeper harmony between chemical principles and biological function that spans multiple scales. What ancient philosophers and more recent naturalists [116] intuited through reasoning, i.e., that geometric forms underlie nature’s physical manifestations, modern coordination chemistry confirms empirically at the atomic scale, particularly in calcium’s triangulated arrangements [117,118].

Ingber has demonstrated that prestress plays a key unifying role in regulating biological responses across multiple length scales [119,120]. Our calcium-centric perspective identifies calcium signaling as the primary molecular mediator of prestress, functioning through its interactions with helical protein domains to rapidly adjust tensional states. This aligns with the concept of the cytoskeleton as a hierarchical tensegrity system whose structural properties at the cellular size scale are determined by local prestress [121].

While Ingber’s work has progressively deepened our understanding of tensegrity across biological scales, his models focus primarily on ATP as the energy source [7,122], integrins as mechanotransducers [123], and hydrogen bonds as tension elements [119,124]. Calcium ions appear only peripherally in these works and mentioned briefly in the context of cellular signaling [125] but not explored in terms of their coordination geometry or potential role in tensegrity structures. Even in his discussions of mechanotransduction pathways, where calcium signaling is mentioned as a downstream effect [119] (p. 811), the specific geometric properties of calcium coordination are not addressed.

### 5.2. Beyond Hydrogen Bonds

The calcium-centric perspective of EMT highlights how calcium’s distinctive coordination geometry—with its triangulated arrangements and flexible coordination numbers—provides a critical molecular mechanism that enables tensegrity principles to manifest across biological scales. We propose that calcium’s octahedral and pentagonal bipyramidal coordination complexes represent fundamental tensegrity units that deserve specific attention, particularly as they create unique tensional networks that differ substantially from those formed by hydrogen bonds.

The distinct geometries of these calcium coordination complexes enable simultaneous interaction with multiple ligands, creating robust and highly connected networks with unique structural properties. In crystalline materials and coordination polymers, calcium polyhedra can be linked by organic ligands to form extended 2D or 3D networks, as exemplified in water-stable calcium coordination networks where seven-coordinated calcium polyhedra connect into layered structures [126]. Similarly, in bone cements, calcium forms coordination networks with phosphoserine, resulting in adhesive crystalline phases that are compositionally tunable and structurally distinct from those formed by hydrogen bonds alone [127].

These calcium-mediated tensional networks differ fundamentally from hydrogen bond networks in several key aspects. While hydrogen bonds create flexible, directional, and relatively weak interactions, calcium coordination generates more rigid and multidirectional networks. The negative electrostatic potential of calcium coordination with carboxyl or oxygen groups in peptides results in strong binding energies and stable complexes that contrast sharply with the more transient nature of hydrogen-bonded networks [128]. These calcium-peptide coordination complexes not only enhance calcium bioavailability but also induce significant conformational changes in target proteins, further highlighting the unique tensional properties of calcium-based networks [128,129].

The functional implications of these distinctive tensional networks extend throughout biological systems, where calcium’s ability to form stable, multidentate complexes underpins its dual role as both signaling ion and structural component. This allows for precise spatial and temporal control that hydrogen bonds alone cannot achieve [130]. In materials science applications, such as bone cements and coordination polymers, these networks impart enhanced mechanical properties and stability unattainable with hydrogen-bonded systems [126,127]. Thus, calcium’s coordination geometry represents a distinct class of tensegrity elements that merits specific consideration in any comprehensive framework of biological architecture.

### 5.3. Calcium Keeps Things Moving: The Dynein-Amic Role of Ca^2+^ in Molecular Motors

While Ingber demonstrated how proteins like dynein motors function as tensegrity structures through a balance of compression-resistant secondary structures and tensional hydrogen bonds [122], our framework complements this by showing how calcium coordination actively regulates these molecular motors. Dynein ATPase activity is strongly modulated by calcium ions and calmodulin, which bind to dynein in a calcium-dependent manner, significantly increasing its enzymatic activity. The LC4 light chain of dynein, a calmodulin family protein with EF-hand domains, acts as a calcium sensor that triggers conformational changes throughout the dynein complex when calcium binds [131].

These calcium-induced conformational changes make the dynein complex more compact, altering its interaction with microtubules and directly affecting its mechanical function [131]. Additionally, in cilia and flagella, calcium and calmodulin mediate the regulation of dynein-driven microtubule sliding, which is fundamental for motility [132]. This calcium-mediated tensegrity perspective offers a meta-understanding of tensegrity organization in biology across eukaryotic organisms, illuminating how calcium’s coordination geometry influences both protein conformation and higher-order mechanical function.

This regulatory principle extends beyond dynein to numerous calcium-binding proteins, particularly evident in EF-hand domains where calcium binding induces dramatic conformational changes that propagate through protein structures [133], and in ECM proteins where calcium coordination influences tissue-level mechanical properties. The common denominator in many of these calcium-mediated tensegrity systems is calmodulin (CaM), which serves as the primary intermediary translating calcium signals into mechanical responses.

While CaM’s role in regulating dynein motors represents one specialized application of calcium-mediated tensegrity, this versatile protein functions as a universal tension-mediator throughout eukaryotic cells, orchestrating mechanical responses across multiple biological scales through its exquisitely calibrated calcium-binding domains.

### 5.4. Calmodulin: Nature’s Tension-Mediator

As previously mentioned, CaM is one of the most important calcium-binding messenger proteins in biology, serving as the primary intracellular calcium sensor in eukaryotic cells. Its significance derives from several key attributes:

Universal Signal Transducer: CaM functions as an essential intermediary that translates calcium signals into cellular responses by undergoing conformational changes upon calcium binding.

Regulatory Hub: It modulates the activity of over 100 different target proteins, influencing a stunningly diverse set of cellular processes, including:

- Muscle contraction
- Neurotransmitter release
- Gene transcription
- Metabolism
- Cell proliferation
- Cytoskeletal dynamics
- Ion channel function
- Memory formation

Structural Exemplar: CaM contains four canonical EF-hand domains, making it the prototypical calcium-sensing protein and a model system for understanding calcium-mediated conformational changes.

Evolutionary Conservation: The calmodulin protein sequence is conserved across all eukaryotes, with human and plant calmodulin sharing approximately 90% sequence identity, underscoring its fundamental biological importance [134].

CaM is ubiquitously distributed throughout the human body:

Universal Cellular Presence: Found in virtually every eukaryotic cell type in humans

Tissue Distribution: Particularly abundant in the following:

- Brain tissue (especially in neurons);
- Cardiac muscle;
- Skeletal muscle;
- Smooth muscle;
- Pancreatic cells;
- Immune cells.

**Subcellular Localization:**

- Cytoplasm (primary location);
- Nucleus (where it regulates transcription factors);
- Associated with plasma membrane (regulating ion channels);
- Bound to the cytoskeleton;
- Present at synaptic junctions in neurons;
- Prevalence and Abundance.

CaM exhibits extraordinary prevalence throughout eukaryotic organisms, including animals, plants, and fungi. In mammalian cells, it typically exists at concentrations of 10–100 μM, constituting approximately 0.1% of total cellular protein in most cell types [135], with even higher concentrations in neural tissue where it can represent up to 1% of total soluble protein and reach concentrations of ~70 μM in brain tissue [136]. Each calmodulin molecule can bind up to four calcium ions through its EF hands with high affinity (Kd ≈ 10^−6^ M), collectively providing significant calcium-buffering capacity within cells. This abundance is maintained through redundant encoding by three different genes in humans (CALM1, CALM2, and CALM3), all producing identical protein products, reflecting strong evolutionary conservation [137,138,139,140,141].

### 5.5. Integration of Frameworks

We propose that calcium’s coordination geometry and its interactions with EF-hand domains demonstrate calcium-mediated tensegrity at every scale. Within the ECM, calcium functions as a key component of a complex adaptive system as defined by Holland [142], orchestrating an ionic, dynamic tensioning mechanism that allows biological structures to adapt bidirectionally. from local to global and vice versa, across scales via prestress, a concept thoroughly developed by Ingber over multiple publications [85,105,121].

Building upon Ingber’s demonstrations of tensegrity principles at multiple biological scales, our calcium-centric perspective extends this framework toward calcium specificity, as its EF hand coordination provides a compelling geometric mechanism for tensegrity-based force transmission. By integrating Ingber’s analyses of cytoskeletal and protein-based tensegrity with our focus on calcium coordination, we provide a narrative synthesis of how geometric principles govern biological organization across all scales.

Our calcium-centric perspective aligns with emerging fascia-centric interpretations of anatomical architecture [143], which similarly emphasize dynamic, tensioned networks that integrate and compartmentalize biological systems. The “fasciategrity” model proposed by Sharkey [144] describes fascia as a continuous, heterogeneous connective tissue network built on tensegrity principles that balance stability and mobility throughout the body, mirroring at the macroscale what our calcium-mediated framework reveals at molecular and cellular levels.

Both perspectives move beyond traditional models that treat structures as isolated parts; instead, they highlight interconnected systems that unify and coordinate function across multiple scales [145]. Just as we demonstrate calcium’s role in mediating tensional integrity from molecular interactions to tissue organization, the architectural approach to fascia emphasizes connective tissue continuity that enables both mobility and stability through specialized yet interconnected compartments [146,147]. The fascial system’s capacity for efficient force transmission and proprioceptive feedback through tensioned networks provides a macroscopic parallel to calcium’s nanoscale role in coordinating protein conformation and cellular mechanics.

### 5.6. Further Clinical Significance and Biomedical Applications

The extensive involvement of EF-hand calcium-binding proteins in human pathophysiology validates calcium coordination as a central pedagogical framework in its own right. These proteins’ presence in major disease processes—from cancer and neurodegeneration to cardiac arrhythmias—demonstrates calcium’s position at the nexus of clinical medicine [148,149,150].

Key examples include:

**a. S100 proteins in disease processes**: The S100 family demonstrates how variations in calcium-binding domains produce wide-ranging pathophysiological effects in cancer, metabolic disorders, and neurological diseases [149,151]. S100A8/S100A9 as inflammation markers connect molecular geometry to diagnostic medicine [152].
**b. Neuroprotective mechanisms**: Calcium-binding proteins like calbindin, calretinin, and parvalbumin influence neuronal vulnerability to neurodegenerative processes, bridging molecular biophysics to clinical neurology [153].
**c. Microbial virulence regulation**: EF-hand proteins regulate virulence factors in pathogens like Pseudomonas aeruginosa, connecting molecular geometry to infectious disease mechanisms [154].

The structural insights provided by EF-hand motifs with their distinctive architecture and calcium-induced conformational changes, offer a mechanistic understanding of signal transduction that is applicable across physiological systems [148,150].

Calcium’s geometric properties hold significant potential for biomedical innovation. Calcium-based materials demonstrate high biocompatibility with tunable nanostructures for bone regeneration [91,155]. Calcium-based Metal–Organic Frameworks (Ca-MOFs) offer promising applications in drug delivery and molecular separations [156]. These applications demonstrate how understanding calcium’s coordination geometry can translate to practical therapeutic advances in biomedical sciences.

## 6. Future Directions

### 6.1. Bridging Calcium Coordination Geometry and Clinical Practice

**Figure 7:**
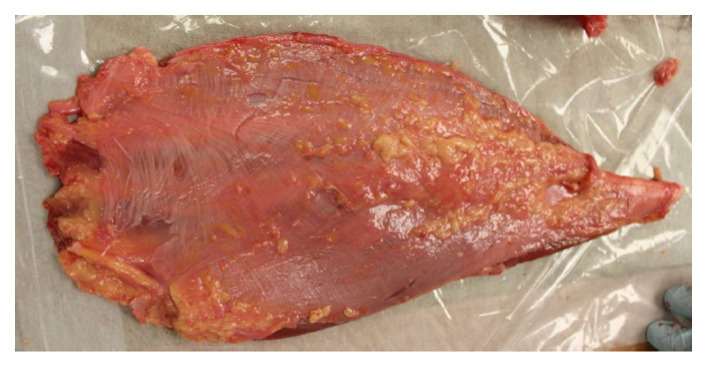
Posterior view of the human quadriceps femoris group, preserved as an integrated fascial-muscular continuum, revealing collagen decussation and the architectural substrate for tensegral force transmission and calcium-responsiveness. This bespoke dissection highlights the posterior fascial interface of the quadriceps muscle group, with the epimysial and intermuscular fascial layers retained en bloc. The image reveals a striking decussation of collagen fibers, suggesting not only mechanical anisotropy but also a directional tuning of load transmission consistent with tensegrity principles. These criss-crossing fibers reflect a living architecture in which prestress and tissue stiffness are not uniform but vary in response to dynamic functional demands.

The integration of coordination chemistry principles with clinical anatomy and fascia research opens unprecedented opportunities for advancing our understanding of human movement and therapeutic intervention. Drawing from the co-authors’ expertise in clinical anatomy education and fascia-based therapeutic approaches, several key research directions emerge that could transform both basic science pedagogy, health professions education, and clinical practice (as noted in Figure 7).

### 6.2. Mechanistic Studies of Ca^2+^ Channels in Fascial Networks

Building on our geometric framework, future investigations should examine the specific roles of Ca^2+^ channels (particularly CaV1.2) within fascial fibroblasts and assess how alterations in Ca^2+^ coordination geometry affect fascial biomechanical properties, including tissue stiffness, load-bearing capacity, and response to manual therapeutic interventions. Recent tendon research demonstrates that Ca^2+^ signaling regulates ECM protein expression, collagen fibrillogenesis, and tissue biomechanics, with enhanced Ca^2+^ signaling leading to tendon hypertrophy and increased stiffness [157,158]. These findings provide a mechanistic foundation for understanding how calcium’s octahedral and pentagonal bipyramidal coordination geometries may directly influence the prestress and mechanical responsiveness that characterize healthy fascial function across multiple scales.

### 6.3. ECM Remodeling Through Calcium-Mediated Tensegrity

Future research should explore how Ca^2+^-dependent coordination pathways influence the expression of ECM proteins (tenascin C, periostin, collagen types) and growth factors (myostatin) within fascial networks. This regulation underpins the structural and functional adaptability of fascia and related connective tissues [159,160]. The geometric specificity of calcium coordination offers a novel lens for understanding how these proteins assemble into the continuous, heterogeneous networks described in fascia-centric anatomical models. This research direction could elucidate the molecular mechanisms underlying the “fasciategrity” principles observed in clinical practice [144].

### 6.4. Coordination Chemistry Effects on Biomechanical Property Modulation via Mesenchyme Healing

The intersection of coordination chemistry and mesenchymal cell biology offers promising avenues for therapeutic intervention in fascial and connective tissue dysfunction. Understanding how metal-ligand coordination influences cellular mechanics and tissue regeneration could provide mechanistic insights into the geometric principles underlying calcium-mediated tensegrity in clinical contexts.

- Modulation of Mesenchymal Cell Biomechanics: Tripeptides and coordination chemistry can significantly enhance the mechanical properties of human mesenchymal stem cells (hMSCs), as demonstrated by a ~2-fold increase in Young’s modulus, which correlates with improved proliferation and wound healing capacity. This suggests that specific chemical cues can direct mesenchymal cell mechanics without inducing unwanted differentiation, supporting tissue regeneration [161].
- Molecular Mechanisms and Biomechanical Restoration: Mesenchymal stem cells (MSCs) can restore impaired biomechanical properties in damaged tissues by regulating collagen content and gene expression. In diabetic skin, MSCs correct biomechanical deficits by modulating miR-29a and increasing collagen, directly impacting tissue strength and healing [162]. Additionally, disruption of mechanotransduction pathways, such as through focal adhesion kinase (FAK) inhibition, can reduce fibrosis and contracture, restore collagen architecture, and improve biomechanical properties in healing tissues, highlighting the interplay between chemical signaling and mechanical outcomes [163,164].
- Bone and Connective Tissue Regeneration: Coordination chemistry strategies, such as gallic acid-calcium grafts, create multifunctional biomaterials that regulate the microenvironment for bone regeneration, influencing inflammation, vascularization, and osteogenic differentiation through pathways like integrin/PI3K/Akt [165]. MSCs also enhance bone healing and biomechanical strength by promoting the release of growth factors (b-FGF, VEGF, OPG) and improving bone mineral density and mechanical parameters, including maximum load and stiffness in fracture models [166].

These findings demonstrate that coordination chemistry, through both direct modulation of mesenchymal cell mechanics and the design of dynamic biomaterials [167] plays a pivotal role in enhancing biomechanical properties and healing outcomes in mesenchymal tissues, supporting the rationale for developing targeted therapies for improved tissue regeneration and functional recovery.

### 6.5. Translational Implications: Drug Development and Biomarker Innovation

The calcium-mediated tensegrity (CMT) framework represents a powerful new paradigm in translational medicine by connecting mechanical forces, calcium signaling, and cellular architecture. The authors aim to bridge these disciplines with fascia science for an integrated pedagogical model. Calcium-mediated tensegrity explains how physical cues are rapidly converted into biochemical signals through mechanosensitive ion channels (such as Piezo1, TRPV4, and stretch-activated channels), leading to calcium influx that triggers downstream signaling pathways remodeling the cytoskeleton and cell junctions [168,169,170].

The tensegrity-based cytoskeletal network distributes and senses mechanical forces, allowing rapid transmission of mechanical signals to the nucleus and other organelles, where they integrate with calcium signaling to orchestrate changes in cell structure, gene expression, and tissue healing [171,172,173,174]. This mechanistic understanding opens pathways for innovative therapies and biomedical protocols that harness or modulate these pathways to improve healing, reduce fibrosis, and enhance tissue regeneration.

#### 6.5.1. Biomarker Development

- Calcium-binding protein conformational markers utilizing conformation-sensitive antibodies that specifically recognize EF-hand domain geometry changes upon Ca^2+^ binding, enabling sensitive detection of disease-associated states such as early-stage malignant melanoma through exosome analysis [175,176].
- Calcium coordination state indicators employing machine learning and computational tools to analyze Ca^2+^ coordination environments in proteins, combined with point-of-care immunosensors for rapid serum Ca^2+^ measurement and real-time diagnostic applications [15,18,177].
- Individual calcium signaling profiles leveraging the diversity in calcium-binding protein conformational responses and coordination states to create personalized diagnostic and treatment stratification approaches, particularly for diseases where calcium signaling is dysregulated [15,18].

#### 6.5.2. Therapeutic Development:

- Calcium-based biomaterials for fascial repair and regeneration utilizing geometric coordination principles to create nanorods, nanowires, nanofilms, and 3D nanoframes that accelerate wound healing by modulating the local microenvironment and promoting orderly tissue repair through calcium’s coordination geometry [168,178].
- Targeted interventions based on EF-hand protein modulation and coordination chemistry to precisely control calcium signaling pathways critical for cell migration, proliferation, and matrix remodeling during fascial healing, enabling therapeutic control over calcium-binding protein conformational states [168].
- Novel therapeutic approaches leveraging calcium coordination biomarkers for precision medicine by assessing individual calcium signaling profiles and protein conformational states to develop personalized treatment strategies that optimize healing outcomes and minimize complications [168,178].

### 6.6. Cross-Scale Research Framework

Future studies should employ the scale-spanning approach demonstrated in Table 1, investigating calcium-mediated tensegrity from atomic coordination to whole-body movement patterns. It is time to integrate scales and disciplines to understand human health within the context of basic sciences. This framework offers unique opportunities for interdisciplinary collaboration between coordination chemists, anatomists, clinicians, and movement scientists. This research agenda represents a paradigm shift from traditional reductionist approaches toward an integrated understanding of how molecular geometry governs human health and movement across all biological scales.

## 7. Conclusions

From the octahedral coordination of a single calcium ion to the triangulated networks of the cytoskeleton, the same architectural principles persist across biological scales. The complementarity between calcium and its binding proteins represents perhaps the most repeated molecular interaction in biology—a silent symphony playing continuously in every living creature.

By reconceptualizing calcium’s role through the lens of geometric principles, we can connect Calcium’s atomic structure to tissue function and the wider complexity ecosystem through a single coherent principle that can be traced across scales to give a unified conceptual framework

## Figures and Tables

**Table 1:** Scale-spanning calcium-mediated tensegrity: from atoms to organisms.

Level of Organization	Approximate Scale	Examples	Calcium-Related Structures, Functions, and Tensegrity Balance
**Subatomic**	10^−15^ m	Electrons, protons, neutrons	Electron orbital interactions that enable calcium’s bonding properties; electron density distributions create tensional balance in coordination sphere
**Atomic**	10^−10^ m	Individual calcium atoms, oxygen atoms	Atomic radius of calcium (114 pm) creates optimal tensional geometry for coordinating 6–8 ligands in balanced triangulated arrangements
**Ionic**	10^−10^ m	Ca^2+^ ions, Mg^2+^ ions, phosphate ions	Electrostatic forces between calcium ions and oxygen ligands establish precise tensional equilibrium in coordination complexes
**Moleular**	10^−9^ m	ATP, glucose, amino acids, water	Calcium-binding loops create tensegrity-based pocket structures; water *molecules* form dynamic tensional networks around calcium ions
**Macromolecular**	10^−8^ m	Proteins (calmodulin, troponin C), DNA, integrins	EF-hand domains function as tensional springs; calcium binding induces balanced *conformational* shifts for signal transduction
**Cytoskeletal**	10^−8^ to 10^−7^ m	Actin filaments, microtubules, intermediate filaments	Calcium regulates tensional states of cytoskeletal networks; modulates compression-tension balance between microtubules and actin filaments
**Supramolecular**	10^−7^ m	Protein complexes, focal adhesions, desmosomes	Calcium-dependent tensional integrity of adhesion complexes; maintains balanced tension across gap junctions and intercellular connections
**Subcellular**	10^−7^ to 10^−6^ m	Mitochondria, endoplasmic reticulum, nucleus	Calcium gradients establish tensional homeostasis between organelles; ER serves as tensional reservoir for calcium-mediated structural stability
**Cellular**	10^−6^ to 10^−4^ m	Neurons, muscle cells, osteoblasts	Calcium waves regulate cellular prestress; modulates tensegrity-based mechanotransduction through cytoskeletal-membrane-nucleus continuum
**Extracellular matrix**	10^−6^–10^−4^ m	Collagen networks, elastin fibers, *proteoglycans*	Calcium-dependent ECM tensional integrity; balances compression-tension elements in fibronectin networks; regulates matrix prestress
**Tissue**	10^−4^–10^−2^ m	Muscle tissue, bone tissue, epithelium	Calcium mediates tissue-level tensional states; maintains tensegrity balance in bone mineralization; coordinates contractile forces across tissues
**Organ**	10^−2^–10^−1^ m	Heart, bones, brain	Coordinated calcium signaling balances tensional forces in cardiac contraction; maintains tensegrity-based structural integrity of organs
**System**	10^−1^–1 m	Skeletal system, nervous system, cardiovascular system	Calcium regulation establishes tensional equilibrium across body systems; coordinates balanced force distribution throughout musculoskeletal network
**Organism**	1 to 2 m	Whole human body	Integrated calcium homeostasis maintains whole-body tensegrity balance; orchestrates tensional harmony across all biological scales

This table illustrates how calcium’s coordination geometry principles manifest across fourteen orders of magnitude in biological organization. Abbreviations: Ca^2+^ = calcium ion; Mg^2+^ = magnesium ion; ATP = adenosine triphosphate; DNA = deoxyribonucleic acid; ECM = extracellular matrix; ER = endoplasmic reticulum.

**Table 2:** First vs. second messenger distinction.

First Messengers	Second Messengers
External ligands (hormones, neurotransmitters)	Internal signaling molecules (Ca^2+^, cAMP, IP_3_)
Bind to cell surface receptors	Released/activated inside the cell
Initiate signaling cascade	Amplify and transmit signals
Cannot cross membrane barriers	Operate within cellular compartments

**Table 3:** Calcium vs. magnesium properties and protein binding [15,22].

Property	Effect on Protein Binding	Comparison to Mg^2+^
Large ionic radius (114 pm)	Enables flexible, high coordination (6–8 sites)	vs. Mg^2+^ (86 pm): rigid, strict 6-coordination
Intermediate charge density (12.6 C/mm^3^)	Matches protein site electrostatics optimally	vs. Mg^2+^ (23.3 C/mm^3^): overly tight binding
Flexible geometry	Supports rapid, reversible interactions	vs. Mg^2+^: static octahedral geometry only
Selectivity over Mg^2+^	Many-body effects and geometric fit favor Ca^2+^	Geometric constraints exclude smaller Mg^2+^

**Table 4:** Calcium signaling pathways in key physiological processes.

Process	Signal Initiation	Protein Conformation Change	Functional Outcome	Key References
**Nerve conduction**	Neurotransmitter/hormone triggers Ca^2+^ influx	Ca^2+^ binds to synaptic proteins	Neurotransmission	[10,23]
**Muscle contraction**	Ca^2+^ release via channels	Ca^2+^ binds to contractile proteins	Muscle contraction	[23,24]
**Cell survival/death**	SOCE, Ca^2+^ channel activation	Ca^2+^ modulates autophagy/apoptosis proteins	Cell fate decisions	[40]

This table illustrates how calcium’s coordination geometry enables diverse physiological responses through protein conformational changes. Abbreviations: Ca^2+^ = calcium ion; SOCE = store-operated calcium entry.

**Table 5:** Major calcium-binding proteins and associated pathologies.

Protein Family	Key Members	Primary Function	Associated Diseases/Disorders	Calcium Binding Domain
**EF-Hand Proteins**				
Calmodulin (CaM)	CaM1, CaM2, CaM3	Universal calcium signal transducer; regulates > 100 target proteins	Cardiac arrhythmias, CPVT, Long QT syndrome, neurodevelopmental disorders, certain cancers	4 EF-hands
Troponin C	cTnC, sTnC	Muscle contraction regulation	Hypertrophic cardiomyopathy, dilated cardiomyopathy, heart failure	4 EF-hands
Calcineurin	CnA, CnB	Phosphatase activity, immune response, cardiac development	Cardiac hypertrophy, immunodeficiency, transplant rejection, Down syndrome	4 EF-hands (in CnB)
S100 proteins	S100B, S100A1-A16	Tissue-specific regulation, inflammation	Alzheimer’s disease, melanoma, psoriasis, rheumatoid arthritis, cancer progression	2 EF-hands
Calbindin	Calbindin-D28k	Calcium buffering in neurons	Parkinson’s disease, epilepsy, Alzheimer’s disease	6 EF-hands
Parvalbumin	α, β isoforms	Calcium buffering in fast-twitch muscles and neurons	ALS, epilepsy, autism spectrum disorders	3 EF-hands
Calretinin	CR	Neuronal calcium buffering	Mesothelioma, colon cancer, Huntington’s disease	6 EF-hands
**Annexins**	Annexins A1-A13	Membrane organization, vesicle trafficking, calcium homeostasis	Cancer, inflammation, autoimmune disorders, thrombosis	Type II calcium binding sites
**C2-Domain Proteins**				
Protein Kinase C	PKC-α, β, γ	Signal transduction, cell proliferation	Cancer, diabetes, cardiovascular disease, Alzheimer’s disease	C2 domain
Synaptotagmins	Syt1-17	Neurotransmitter release, membrane fusion	Epilepsy, neurodevelopmental disorders, psychiatric disorders	C2 domains
**Calcium Channels**				
Voltage-gated Ca^2+^ channels	CaV1.1-1.4, CaV2.1-2.3, CaV3.1-3.3	Calcium influx, excitation-contraction coupling	Migraine, epilepsy, ataxia, hypokalemic periodic paralysis, Timothy syndrome	EF-hand-like domains
Ryanodine receptors	RyR1, RyR2, RyR3	Calcium release from SR/ER	Malignant hyperthermia, central core disease, CPVT, heart failure	EF-hand-like domains
IP_3_ receptors	IP_3_R1, IP_3_R2, IP_3_R3	Calcium release from ER	Spinocerebellar ataxia, Alzheimer’s disease, Huntington’s disease	EF-hand-like domains
STIM/Orai	STIM1, STIM2, Orai1-3	Store-operated calcium entry	SCID, Stormorken syndrome, tubular aggregate myopathy, York platelet syndrome	EF-hand (in STIM)
TRP channels	TRPV, TRPC, TRPM, TRPA, TRPP, TRPML	Sensory transduction, calcium homeostasis	Polycystic kidney disease, mucolipidosis type IV, pain syndromes, cancer	Various
**ECM Proteins**				
Fibrillin	Fibrillin-1, -2, -3	ECM structural organization, growth factor regulation	Marfan syndrome, congenital contractural arachnodactyly	cbEGF domains
Matrix Gla Protein (MGP)	MGP	Inhibits tissue calcification	Vascular calcification, Keutel syndrome	Gla domains
BM-40/SPARC/Osteonectin	SPARC	Cell–matrix interactions, tissue remodeling	Osteogenesis imperfecta, cataracts, cancer progression	EF-hand pair
**Calcium Sensing Proteins**				
Calcium-sensing receptor	CaSR	Extracellular calcium sensing	Familial hypocalciuric hypercalcemia, autosomal dominant hypocalcemia, hyperparathyroidism	Venus flytrap domain
Neuronal calcium sensors	NCS-1, VILIPs, KChIPs, GCAPs	Neuronal calcium signaling	Schizophrenia, bipolar disorder, retinal degeneration	EF-hands
**Calcium Buffers/Transporters**				
Calsequestrin	CASQ1, CASQ2	SR calcium storage	Catecholaminergic polymorphic ventricular tachycardia (CPVT), malignant hyperthermia	Acidic domains
PMCA pumps	PMCA1-4	Calcium extrusion from cells	Hearing loss, neurological disorders, cardiovascular disease	Acidic regions
SERCA pumps	SERCA1-3	Calcium sequestration into SR/ER	Brody disease, Darier disease, heart failure	Transmembrane domains
NCX exchangers	NCX1-3	Sodium-calcium exchange	Cardiac arrhythmias, heart failure, hypertension	α-repeats

**Abbreviations**: CPVT: Catecholaminergic Polymorphic Ventricular Tachycardia; ALS: Amyotrophic Lateral Sclerosis; SR: Sarcoplasmic Reticulum; ER: Endoplasmic Reticulum; SCID: Severe Combined Immunodeficiency; ECM: Extracellular Matrix; cbEGF: Calcium-binding Epidermal Growth Factor; PMCA: Plasma Membrane Calcium ATPase; SERCA: Sarco/Endoplasmic Reticulum Calcium ATPase; NCX: Sodium–Calcium Exchanger.

## Data Availability

No new data were created or analyzed in this study. Data sharing is not applicable to this article.
